# A curated gluten protein sequence database to support development of proteomics methods for determination of gluten in gluten-free foods

**DOI:** 10.1016/j.jprot.2017.03.026

**Published:** 2017-06-23

**Authors:** Sophie Bromilow, Lee A. Gethings, Mike Buckley, Mike Bromley, Peter R. Shewry, James I. Langridge, E.N. Clare Mills

**Affiliations:** a School of Biological Sciences, Manchester Institute of Biotechnology, Manchester Academic Health Sciences Centre, University of Manchester, M17DN, UK; b School of Earth and Environmental Sciences, Manchester Institute of Biotechnology, University of Manchester, M17DN, UK; c Waters Corporation, Stamford Avenue, Altrincham Road, Wilmslow SK9 4AX, UK; d Genon Laboratories Limited, Cragg Vale, Halifax, UK; e Rothamsted Research, Harpenden, UK

**Keywords:** AO, Allergen Online database, BP, BioPep database, BLAST, basic local alignment search tool, cDNA, complementary DNA, FALCPA, Food Allergen Labelling and Consumer Protection Act, HMW, high molecular weight, IM, ion mobility, LMW, low molecular weight, ppm, parts per million, *Triticum aestivum*, Gluten, Celiac toxic motif, Database, Proteomics

## Abstract

The unique physiochemical properties of wheat gluten enable a diverse range of food products to be manufactured. However, gluten triggers coeliac disease, a condition which is treated using a gluten-free diet. Analytical methods are required to confirm if foods are gluten-free, but current immunoassay-based methods can unreliable and proteomic methods offer an alternative but require comprehensive and well annotated sequence databases which are lacking for gluten. A manually a curated database (GluPro V1.0) of gluten proteins, comprising 630 discrete unique full length protein sequences has been compiled. It is representative of the different types of gliadin and glutenin components found in gluten. An in silico comparison of their coeliac toxicity was undertaken by analysing the distribution of coeliac toxic motifs. This demonstrated that whilst the α-gliadin proteins contained more toxic motifs, these were distributed across all gluten protein sub-types. Comparison of annotations observed using a discovery proteomics dataset acquired using ion mobility MS/MS showed that more reliable identifications were obtained using the GluPro V1.0 database compared to the complete reviewed Viridiplantae database. This highlights the value of a curated sequence database specifically designed to support the proteomic workflows and the development of methods to detect and quantify gluten.

**Significance:**

We have constructed the first manually curated open-source wheat gluten protein sequence database (GluPro V1.0) in a FASTA format to support the application of proteomic methods for gluten protein detection and quantification. We have also analysed the manually verified sequences to give the first comprehensive overview of the distribution of sequences able to elicit a reaction in coeliac disease, the prevalent form of gluten intolerance. Provision of this database will improve the reliability of gluten protein identification by proteomic analysis, and aid the development of targeted mass spectrometry methods in line with Codex Alimentarius Commission requirements for foods designed to meet the needs of gluten intolerant individuals.

## Highlights

• A curated database comprising 630 gluten protein sequences has been compiled.
• Sequences represent the gliadin and glutenin protein species found in gluten.
• Coeliac toxic motifs were distributed across all gluten protein sub-types.
• It gave more reliable identifications than the reviewed Viridiplantae database.

## Graphical abstract

**Figure:**
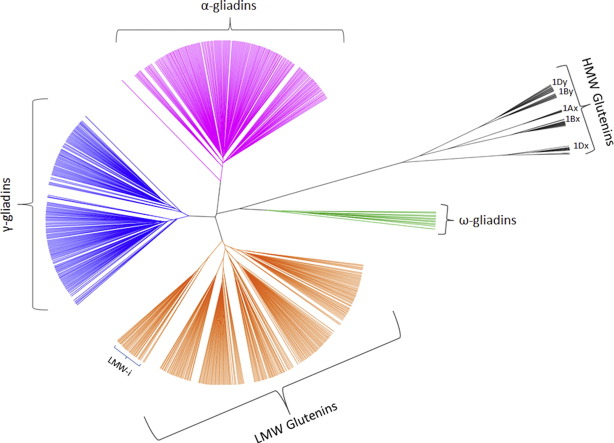

## 1 Introduction

Wheat, the most important food crop worldwide, can be used in the manufacture of a diverse range of food products due to the unique physiochemical properties of gluten. Gluten comprises the major storage proteins of wheat and related cereals such as rye and barley; these proteins are defined as prolamins based on their high contents of the amino acids proline and glutamine which respectively comprise 15% and 35% of the total amino acid composition [1]. Wheat gluten accounts for 70–80% of the grain protein and comprises two fractions which are distinguished based on their solubility in aqueous alcohol mixtures: the alcohol-soluble monomeric gliadin proteins and the alcohol-insoluble polymeric glutenin proteins [1]. The gliadins are traditionally classified as prolamins based on their solubility and high contents of the amino acids proline and glutamine. However, we now know that the glutenin subunits are also soluble in aqueous alcohols after reduction of inter-chain disulphide bonds and that the gliadin and glutenin subunits are closely related in their amino acid sequences. It is therefore usual to classify the gliadins, glutenin subunits and related proteins in other cereals as prolamins.

The gliadins are further classified into α-,γ- and ω-gliadin fractions, whilst reduction of the inter-chain disulphide bonds that stabilise the glutenin polymers allows the component subunits to be divided into high molecular weight (HMW) and low molecular weight (LMW) groups [1], [2].

Gluten is responsible for eliciting coeliac disease, a non-IgE mediated food intolerance, affecting about 1% of the global population for which there is currently no cure [3], [4]. Consequently patients must adhere to a gluten-free diet, the Codex Standard stating that gluten-free foods must contain less than 20 ppm of gluten from wheat, barley, rye and oats and their crossbreeds [5]. This broad definition has been implemented worldwide, although variations occur and the United States recently excluded oats from the definition of gluten [6]. The Codex Standard also recommends using immunobased methods (or alternative methods) that are able to achieve appropriate sensitivity and specificity for the detection and quantification of gluten with a 10 ppm limit of detection [5]. Consequently the current gold standard method for detection of gluten is enzyme linked immunosorbent assay (ELISA) although the high sequence similarity between proteins in members of the grass family, can result in cross-reaction with proteins in contaminating seeds of wild grasses [7]. The extraction efficiency of the ELISA compatible buffer has also been questioned as it can result in misrepresentation of the total gluten content of samples [8]. Protein sequencing by mass spectrometry (i.e., proteomics) has the potential to offer an alternative, complementary method to determine gluten proteins in foods but for the methodology to become fully validated and accepted it must also overcome similar challenges to immunoassay methods, such as effective extraction of samples and the identification of peptide targets with the requisite specificity.

Although proteomic methods for detecting and quantifying mammalian proteins have advanced over the last decade [9], [10], [11], the detection and quantification of plant proteins has encountered many difficulties [12]. Central to a mass spectrometry (MS)-based approach is the requirement for either a functionally annotated genome of the species under investigation or a well curated set of sequences from other sources such as UniProt. The lack of reliable curated sequence databases for plant species is one of the major hurdles limiting progress in this field [13]; out of ~ 35,000 cultivated plant species only 37 have sequenced and functionally annotated genomes, with a major focus on the model plant species *Arabidopsis thaliana* [13], [14]. UniProt has focussed on the curation of plant-derived protein sequences from *A. thaliana* and *Oryza sativa* [15] and the complete reviewed Viridiplantae database in UniProt contains just over 37,000 sequences. The poor coverage of non-model plant species has led many researchers to develop manually curated databases to support protein identification [16], [17]. Furthermore, although a number of full plant genome sequences are available and others are currently being determined, they often pose challenges due to their large size and complexity, including the fact that many, including major crops such as wheat (*triticum*), oilseed rape (*canola*) and sugar cane (*Saccharum officinarum*), are polyploid. The latter in particular poses problems for sequence annotation and database curation. Apart from limiting progress in the application of proteomics to plant science, the lack of appropriate databases also limits the application of proteomic methods in food science, where it has potential applications in authenticity testing [18], [19] and for the determination of allergens, including gluten, in foods [7], [20], [21], [22], [23]. Curated sequence databases for food allergens include Allergen Online and the ProPepper database [24], the latter being the largest collection of prolamin proteins (which include wheat gluten) from cultivated cereals in the grass family (Poaceae). However, these have not been tailored to use in proteomic workflows.

The primary aim of this research was to construct a curated custom wheat gluten protein database (GluPro V1.0; hereafter referred to as ‘GluPro’), in the FASTA format which is based on full length cDNA sequences exclusively from hexaploid bread wheat (*Triticum aestivum*). Hexaploid wheat was chosen as it is representative of the types of the coeliac-toxic gliadins encoded by the A and B genomes of both hexaploid wheat species and tetraploid durum wheat [25] whilst the d-genome γ-gliadins are particularly potent triggers of coeliac disease [26]. In addition the gluten proteins encoded by the D genome make an important contribution to the viscoelastic properties of gluten required for bread-making quality. The sequences in this database have been classified into the different types of proteins that make up the gluten fraction of wheat and have been interrogated to determine the distribution of sequence motifs that have been shown to be coeliac toxic [27]. The GluPro database has been applied to mining untargeted mass spectrometry data sets obtained using ion mobility mass spectrometry using data independent acquisition methods and compared with the reviewed UniProt Viridiplantae database with regards gluten protein sequence annotations.

## 2 Materials and methods

### 2.1 Identification of gluten protein accessions in UniProt

The complete reviewed Viridiplantae database (37,577 sequences from all plant species 14.05.2015) was downloaded from UniProt, in FASTA format. Gluten proteins were manually identified using protein descriptions containing the terms “gluten”, “gliadin”, “glutenin”, “avenin” and “prolamins”. The identified proteins were then searched against the GluPro database to identify those common and unique to each sequence database.

### 2.2 GluPro database construction and curation

**Fig. S1:**
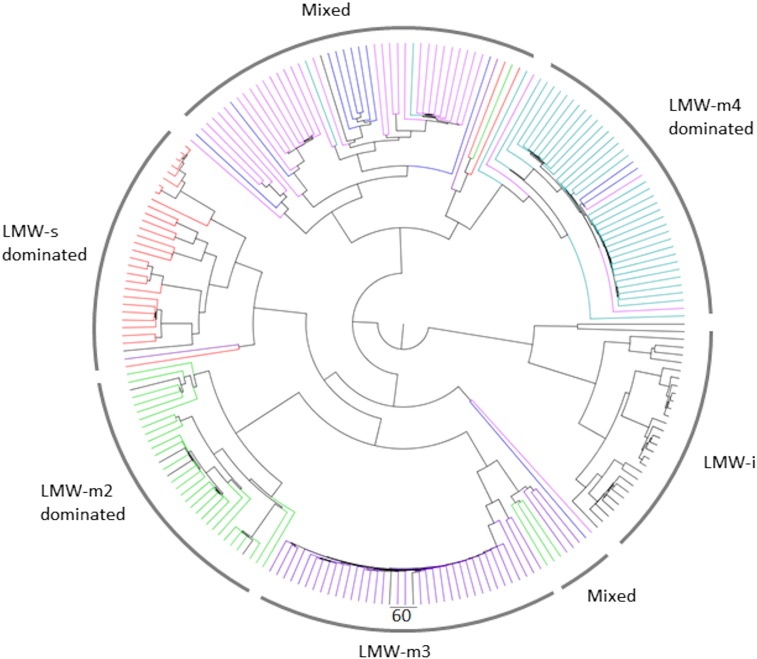
Phylogenetic tree of the LMW glutenins, coloured by N-terminal sequence classifications. With the distinct LMW-i subgroup shown in black, LMW-s in red and the LMW-m1, -m2, -m3, -m4 and -5 subgroups shown in blue, green, purple, teal and pink respectively.

A well curated and previously verified set of gluten protein sequences [28] representative of each of the five different gluten protein subgroups (α-, γ-, ω-gliadins, LMW and HMW glutenin subunits) was used to underpin the development of the GluPro database. Each of the five gluten protein subgroup databases was developed independently and then finally merged into a single database using an approach summarised in Supplementary Fig. S1. Using the seed sequences the unreviewed UniProt database was mined [29], [30]. The UniProt accession for each verified sequence was BLAST (basic logic algorithm search tool) searched against the entire UniProt database, and the search parameters set to show 1000 results and filtered on the criterion of only sequences from *T. aestivum*. The resulting sequences from each BLAST search were downloaded and saved and combined to provide a single file per subgroup prior to curation. Redundant sequences were removed using DB toolkit [31] and the remaining sequences aligned using Clustal Omega and saved as a MSF file. These were viewed in Jalview [32] to allow multiple sequence alignment analysis with the sequence coloured by percentage identity to show regions of high homology. At this stage sequences were manually interrogated to remove incomplete and incorrectly annotated sequences. This was continued until a set of unique full length sequences remained for each of the five subgroups. The five resulting curated sequence sets were combined to form the complete GluPro database prior to re-checking for duplicate sequences using the DB toolkit. The resulting list of the verified UniProt sequence accessions and the corresponding number of downloaded sequences for each accession can be found in the Supplementary Table S1 and downloaded in FASTA format (http://dx.doi.org/10.15127/1.308603).

### 2.3 Phylogenetic analysis

Phylogenetic analysis was carried out on the five subgroup sequence databases and on the complete GluPro database using neighbour-joining with the BLOSUM62 algorithm in Jalview [32] and the tree viewed and manipulated in FigTree [33]. These were used to illustrate relationships between and within the five subgroups in the complete and subgroup databases; a full description of the further classifications can be found in Supplementary Table S2.

### 2.4 Coeliac toxic motifs

The coeliac toxicity of the sequences in the GluPro database was investigated using four in silico measurements utilising two online databases: 1) the Allergen Online Celiac Disease – Novel Risk Assessment Tool (AO) [34], an online database containing 1016 coeliac toxic motifs (although many of which are fragments of others) with an exact peptide function that allows these peptides to be mapped, and 2) BIOPEP (BP) [35], an online sequence database including proteins, bioactive peptides and allergenic proteins with their epitopes which also includes tetrapeptide motifs (QQQP, PSQQ, QQPY and QYPY) that are repeatedly observed in gliadin proteins, particularly α-gliadin. The four measure of coeliac toxicity where as follows:

Unique motif measurement: Each sequence was searched against the AO coeliac toxic database using the exact peptide match function, and the number of unique coeliac toxic motif matches recorded.
Density of motifs: The density of coeliac toxic motifs was calculated by dividing the number of coeliac toxic motifs by the number of amino acids in the sequence.
Percentage (%) sequence coverage: The AO list of 1016 coeliac toxic motifs was used alongside protein coverage software [36] to calculate the percentage (%) of each GluPro sequence covered by coeliac toxic motifs.
A-value: The GluPro database α-gliadin protein sequences were put into the BP database and the A-value (number of coeliac toxic fragments divided by the number of amino acids in the sequence) calculated and recorded. This measurement was compared to the value obtained for the number of unique coeliac toxic motifs (Measurement 1) to identify correlations. This specific measurement of the coeliac toxicity of the α-gliadins was used because of the bias in occurrence of the tetrapeptides.

The 1016 coeliac toxic motifs downloaded from the AO database were manually mapped onto identified proteins from the MS data searched against the GluPro database, to investigate how the observed peptides cover the hazardous regions of the gluten proteins.

#### 2.4.1 IgE epitope mapping

An in-house collated set of 55 IgE epitopes (Supplementary Table S3) taken from previously published literature [36], [37], [38], [39], [40] was mapped to each sequence in the GluPro database.

#### 2.4.2 Protein mass spectrometry

Gluten proteins were extracted from crushed bread wheat (*T. aestivum* cv Hereward) grains using an extraction buffer comprised of Rapigest (0.02% *w*/*v*), DTT (50 mM) and Tris-HCl (50 mM, pH 8.8). Extracted proteins were reduced, alkylated and digested using chymotrypsin and subject to liquid chromatography coupled with tandem mass spectrometry (LC-MS/MS) analysis as described [42]. LC-MS/MS analysis was carried out using a SYNAPT G2-Si QTOF (quadrupole time of flight) (Waters, Wilmslow, UK) with incorporated ion mobility. Three biological replicates (single seeds) were analysed in triplicate. The three seeds were selected after a sample of seeds (n = 100) were weighed and plump seeds representative of the average weight of total seed sample was selected. Aliquots (1 μL) of digested sample extracts were chromatographically separated on an M-class ACQUITY UPLC system (Waters, Wilmslow, UK) using a NanoEase 1.8 μm HSS T3 C18 (75 μm × 150 mm) column (Waters) using a linear gradient (flow rate 300 nL/min) from 3 to 40% (v/v) solvent B over 90 min. The mobile phases consisted of solvent A (0.1% (v/v) formic acid/99.9% (v/v) water) and solvent B (0.1% (v/v) formic acid/99.9% (v/v) acetonitrile). Data were acquired using an ion mobility assisted data independent analysis (IM DIA) mode of acquisition. MS analysis was performed in positive ion mode over the mass range *m*/*z* 50–2000 with a 0.5 s spectral acquisition time. One cycle of low and elevated energy data was acquired every 1 s [42]. Data were processed using Progenesis QI for proteomics V2.0 [16] and searched against each database (GluPro V1.0 and reviewed Viridiplantae) individually. The resulting protein identifications were compared.

## 3 Results

### 3.1 GluPro database development and characterisation

Verified sequences representing the HMW and LMW subunits of glutenin and the α-, β-, and ω-gliadins [28] were used to mine the unreviewed UniProt database and retrieved over 24,000 sequences (Supplementary Table S1). With the exception of the ω-gliadin sequences, around 90–95% of the original sequences downloaded were redundant and removed in the initial step. Then 65–80% of the remaining non-redundant sequences were removed as either being partial sequences or being mis-assigned to *T. aestivum*, coming instead from other plant species. For example, UniProt sequence #A3RF25 was removed from the HMW glutenin subunit sequence set (288 sequences) as it is a fragment consisting of only 386 residues whereas UniProt sequence #Q6UJY7 was removed because it originates from the tetraploid wheat species *T. turgidum*. In contrast, fewer duplicate sequences were downloaded for the ω-gliadins but many more were either incomplete or mis-assigned. The final database (GluPro V1.0) was composed of 630 sequences (Supplementary Table S2), made up of 55 sequences belonging to the HMW subunits and 224 sequences corresponding to the LMW subunits of glutenin, together with 185 α-gliadin, 154 γ-gliadin and 12 ω-gliadin sequences (Table 1). This distribution is consistent with the proportions of the different types of gluten proteins observed by 2D PAGE profiling [43].

**Table 1:** GluPro database composition: Sequences identified by BLAST searching against seed sequences had redundant sequences removed automatically using the DB Toolkit and the remaining sequences were verified manually.

Gluten subgroup	Number of sequences		
	BLAST search	Non-redundant sequences (%)	Verified sequences (%)
HMW	5142	288 (5.6)	55 (1.1)
LMW	8966	637 (7.1)	224 (2.5)
α-Gliadin	4601	511 (11.1)	185 (4.0)
γ-Gliadin	5500	621 (11.2)	154 (2.8)
ω-Gliadin	414	347 (84)	12 (2.3)
Total	24,623	2404	630

**Fig. 1:**
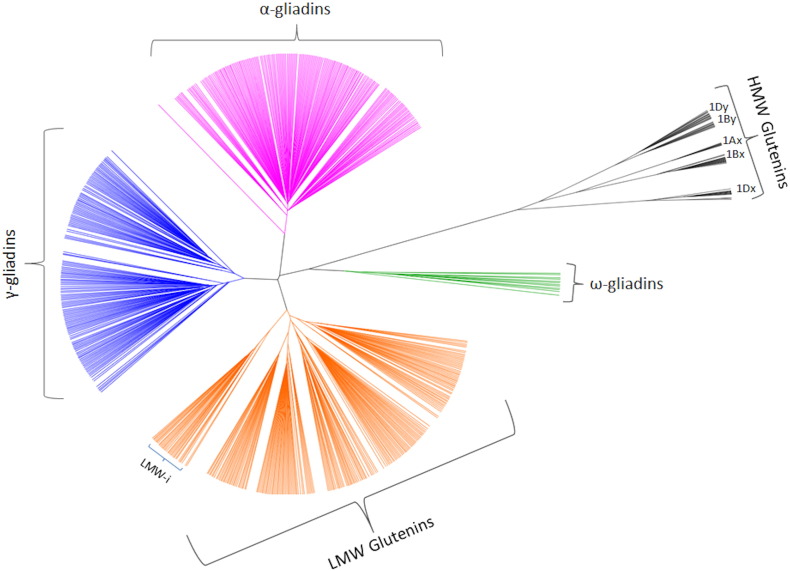
Phylogenetic tree for the complete curated wheat gluten database GluPro. The tree was created using the BLOSUM 62 algorithm in Jalview and then manipulated in FigTree. The three prolamin groups α-, γ- and ω-gliadins and the HMW and LMW glutenins are coloured pink, blue, green, grey and orange respectively. Where further sub-classifications cluster distinctly on the tree these are labelled, this is observed for the LMW-i and LMW-s subgroups of the LMW glutenins and the Ax, Bx, By, Dx and Dy subgroups of the HMW glutenins.

Phylogenetic analysis of the aligned sequences from the GluPro database revealed a clustering as expected into the glutenin and gliadin subgroups (HMW and LMW subunits of glutenin and α-,γ- and ω- gliadins respectively) (Fig. 1). The α- and γ-gliadin proteins are highly homologous with an average sequence identity of 86.89% and are closely related to the LMW subunits of glutenin. This is consistent with the previous suggestion that the LMW subunits of glutenin are derived from an ancestral protein common to the γ-gliadins, being distinguished mainly by the presence of cysteine residues which form intermolecular disulphide bonds, enabling them to form disulphide-linked polymers. The ω-gliadins differ in that their primary sequences comprise essentially only repeated peptide motifs and hence lie on a separate branch on the tree to the other gliadins and LMW subunits of glutenin. The HMW subunits of glutenin also differ, particularly in the presence of a longer domain of repetitive sequences which are not related to those in other gluten proteins.

**Fig. 2:**
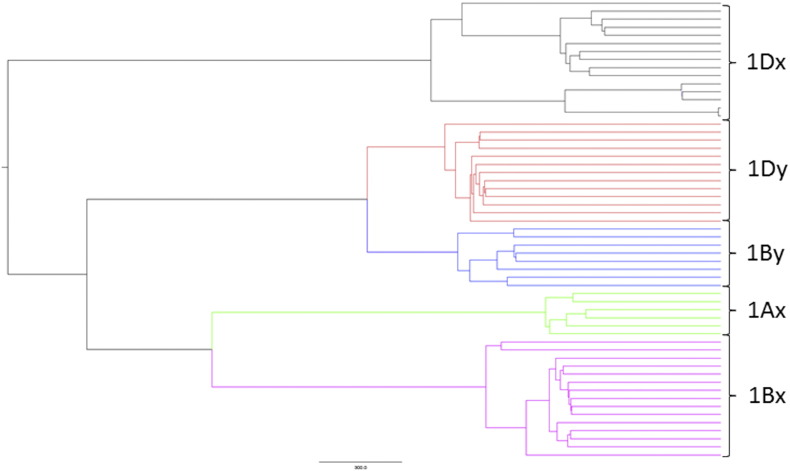
A phylogenetic tree of the 55 HMW subunits of glutenin sequences in the GluPro v1.0 database. Sequence alignments were created using the BLOSUM 62 algorithm. Branches are labelled relating to the encoding of subunits on chromosome 1 of the A, B and D genomes of *Triticum aestivum* and the classification of subunits based on SDS-PAGE electrophoresis into x-type and y-type subunits.

Detailed analysis of the HMW subunits of glutenin showed clustering into five groups corresponding to the x-type subunits encoded by chromosomes 1A, 1B and 1D and the y-type subunits encoded by chromosomes 1B and 1D (1A y-type subunits not being expressed in bread wheat (Fig. 2)) [28]. The same alignment was observed in the master phylogenetic tree derived from the total database (Fig. 1) and a tree built using the HMW subunits sub-database alone.

The sequences in the sub-database comprising LMW subunits of glutenin could also be further classified into seven distinct groups using a previously described approach based on their N-terminal amino acid sequences [43], [44]. Thirty of the sequences correspond to the LMW-i group which differs from the other groups in that it lacks a non-repetitive N-terminal sequence, starting with the repetitive domain. The LMW-s group comprised 31 sequences, with the remaining 163 sequences corresponding to the closely related LMW-m1, m2, m3, m4 and -m5 groups which are highly homologous sequences and may differ in only a single amino acid at the start of the N-terminal domain (Supplementary Table S4). This classification into seven groups based on N-terminal sequence was not observed in the phylogenetic analysis for either the LMW glutenin subgroup database (Supplementary Fig. S1) or the complete GluPro database. However in both analyses the LMW-i formed a distinct cluster from the other subgroups (Fig. 1).

Phylogenetic analysis of the γ-gliadin sequences using the sub-database revealed four major and two minor groups (Supplementary Fig. S3). Group 1 comprises 31 γ-gliadin sequences distinguished by the presence of a characteristic nonapeptide (PQQPFQLQPQ) at approximately amino acid residues 105–140. The second group comprises 24 sequences characterised by a motif QPQQQFIQPQQ starting at residue 90 whilst Group 3 consists of 61 sequences characterised by the 16 amino acid residue motif, QQPQQQFPQQPQQPFP, found in the repetitive domain. The fourth major group comprises 25 sequences which are characterised by a peptide sequence QQTYPHQPQQQFPQTQQPQQPFPQP at residue 90. The two minor groups (Groups 5 and 6 comprising three and 10 sequences, respectively) contained truncated partial repetitive domains compared to the other four groups. These six groups did not cluster distinctly when the entire GluPro database was aligned, with only two distinct groups being observed instead (Fig. 1). An analysis of the number of cysteine residues (7, 8 or 9) showed no clear relationship with the clustering observed in either the master phylogenetic tree or the tree created with the γ-gliadin sub-database.

Following previously established categorisation, the α-gliadin sequences were classified into three groups based on the presence or absence of four immunodominant coeliac toxic peptides that originate from the A, B or D genomes [24], [45]. These immunodominant motifs, which are recognised by the majority of CD patients, are Glia-α9: PFPQPQLPY, Glia-α20: PFRPQQPYPQ, Glia-α2: PQPQLPYPQPQPQLPY and Glia-α: QGSFQPSQQ. However, this method for classification is ambiguous as there is overlap between the gene types with regard to the expected presence or absence of the immunodominant peptides. The clustering of the α-gliadin sequences observed in the phylogenetic tree derived from the α-gliadin sub-database followed the genetic origin of the different subunits (Supplementary Fig. S4). When a protein matched multiple classifications the clustering on the phylogenetic tree was used to confirm the origin of the sequence. The largest proportion of the protein sequences (89) was attributed to the D genome, with 61 and 36 proteins attributed to the A and B genomes, respectively (Supplementary Fig. S4). This was in contrast to the homology clustering observed on the phylogenetic tree for the entire GluPro database which showed one main group, with three smaller groups.

Although only a small proportion of the database is comprised of ω-gliadin sequences these could be further classified based on their N-terminal sequences into those that contain SRLL or AREL/ARQL, which are respectively termed ω-5 and ω-1/2 [47], [48]; of the 12 ω-gliadin sequences in the GluPro database only one sequence was classified as ω-5, with the remaining 11 all possessing AREL or ARQL N-terminal sequences.

### 3.2 Mapping of coeliac toxic motifs across gluten sequences in the GluPro database

**Fig. 3:**
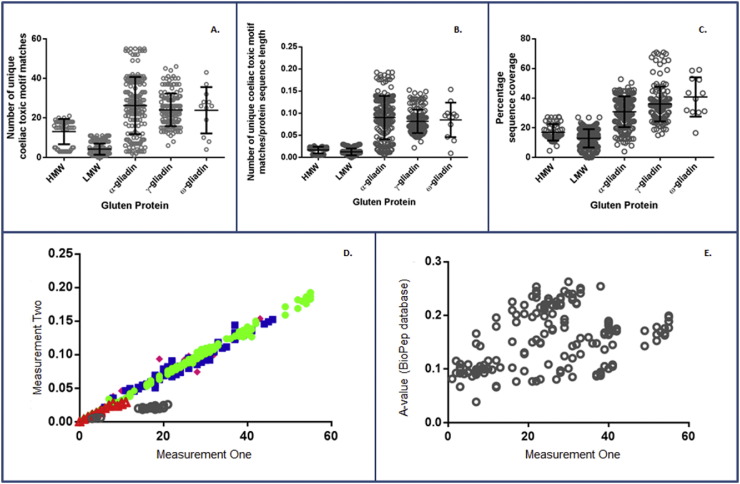
Distribution of coeliac toxic motifs across the GluPro V.1 Database. Each sequence is represented by an individual grey circle, across the five groups of gluten proteins in panels A–C. In Panel D the five groups of gluten proteins are represented by different coloured shapes as follows: grey circle = HMW glutenins, red triangles = LMW glutenins, green dot = alpha gliadins, blue square = gamma gliadins and pink diamond = omega gliadins. A: The number of unique coeliac toxic motifs from Allergen Online database matched to each sequence in the GluPro database. B: The number of unique coeliac toxic motifs identified per protein (from A) was divided by the length of the protein sequence; C: percentage of protein sequence coverage by coeliac toxic motifs calculated by the Protein Coverage Summarizer software; D: a plot of number of unique coeliac toxic motifs (measurement one, A) vs the motif density per sequence (measurement two, B); E: a plot of the A-values calculated by the BioPep database against the number of unique coeliac toxic motifs per sequence (measurement one A) for the α-gliadins.

Initially the number of unique coeliac toxic motifs per protein sequence in the GluPro database was defined using the AO database using the exact peptide match function (Fig. 3a). The 1016 coeliac toxic motifs that were identified were then downloaded and further interrogated. Although the individual gliadin proteins differ widely in their contents of coeliac toxic motifs, the mean numbers per protein were similar for the α-, γ- and ω-gliadins with 26, 24 and 23 unique coeliac toxic motifs per protein species, respectively. However, whereas 19% of the α-gliadin sequences contained > 40 unique coeliac toxic motifs, only 6.5% of the γ-gliadin and 8.3% of the ω-gliadin protein sequences have > 40 unique motifs.

In contrast, the LMW subunits of glutenin had the lowest contents of coeliac toxic motifs with a mean of only four unique motifs per sequence (Fig. 3). The HMW subunits of glutenin had a mean of 13 unique coeliac toxic motifs per sequence (Fig. 3); however, the proteins were divided into two distinct groups. The first had a low content of coeliac toxic motifs, with a maximum of five unique coeliac toxic motifs identified per sequence similar to the LMW subunits of glutenins; these correspond to y-type subunits encoded by the D genome (1Dy). The second group had a higher content of coeliac toxic motifs, similar to the numbers in the gliadin proteins, between 14 and 21 coeliac toxic motifs per sequence.

Since the different types of prolamin vary in length, the density of coeliac toxic motifs was also calculated (Fig. 2b). This showed that the HMW subunits of glutenin had the lowest density of coeliac toxic motifs, consistent with their being the largest gluten proteins (containing > 800 amino acids per sequence compared to the other gluten proteins which all contain ~ 300 amino acids per sequence). A comparison between the number of unique motifs versus the motif density (Fig. 2d) showed a direct correlation for all gluten protein sequence types apart from some HMW subunits of glutenin for which the number of coeliac toxic motifs was independent of the length of the sequence.

The percentage sequence coverage by coeliac toxic motifs (Fig. 2c) shows that the gliadins had the highest content of toxic motifs, accounting for 40.8% of the sequence in the ω-gliadins, followed by 36% in the γ-gliadins and 30.8% in the α-gliadins. Conversely, the LMW glutenins had the lowest potential coeliac toxicity by this measure.

Finally, the GluPro database α-gliadin protein sequences were entered into the BP database and the A-value calculated (i.e. the proportion of the entire sequence that is coeliac toxic). The A-values obtained from the BP database were then compared with the number of unique coeliac toxic motifs matched for the α-gliadin sequences to assess any correlation between different coeliac toxic motif databases (Fig. 2e), but no obvious correlation was observed between the two measures.

### 3.3 Application of the GluPro database to gluten proteomics

The utility of the GluPro database for gluten protein profiling using mass spectrometry was then compared with using only gluten proteins annotated in UniProt. An approach using IM-MS/MS was employed, since we have previously found that although other platforms are also highly effective in identifying gluten proteins, this approach provides a greater number of annotations as it is more effective at sequencing of peptides > 15 residues in length [42]. Of the 37,577 (14.05.2015) reviewed sequences from UniProt ascribed to Viridiplantae, 0.13% (49) were identified as corresponding to gluten proteins using the protein description terminology. Of the 49 identified gluten protein sequences, 44% (22 protein sequence accessions) corresponded to proteins related to the avenins of oats. These were not present in the GluPro database which contains only wheat gluten protein sequences. The remaining 27 sequences were searched against the GluPro database showing that 18 were common across both databases. Investigation of the remaining nine sequences not observed in the GluPro database showed that six sequences were fragments, one (UniProt accession #O24463) is a “prolamin-binding” protein that is not a gluten protein and does not interact with prolamins [49], whilst the remaining two sequences were considered duplicates of sequences with different accession numbers that were removed during the GluPro database curation process.

**Fig. 4:**
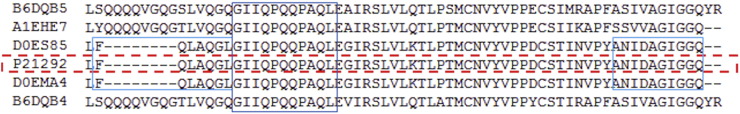
Partial sequence alignment of multiple gamma gliadins showing the occurrence of peptides deemed unique when Viridiplantae database was used to search MS data. Peptides (FQLAQGL, GIIQPQQPAQL and ANIDAGIGGQ, indicated by the blue solid-lined boxes) deemed unique to P21292 (highlighted in the red dashed-lined box), are present in multiple other gamma gliadins (Uniprot accessions: B6DQB5, A1EHE7, D0ES85, D0EMA4, B6DQB4).

When the mass spectrometry data were searched against the GluPro database 39 protein species were identified with at least one unique peptide, and 13 with at least three unique peptides. Of the 44 gluten proteins presents in the Viridiplantae database, 27% (12 protein accessions) were identified in the samples with at least one unique peptide, with only 9% (four protein accessions corresponding to UniProt Accessions #P21292, #P08489, P08488 and #P10386) being identified with more than three unique peptides. Interestingly, on further inspection of these four gluten proteins, all are present in the GluPro database but none were identified in the processed mass spectrometry data searched against the GluPro database. This can be explained by the fact that none of the peptides deemed “unique” when using the Viridiplantae database for searching were considered unique when using the GluPro database. For example, three unique peptides where identified for UniProt accession #P21292 using the Viridiplantae database (FQLAQGL (exemplar MS/MS spectra is shown Supplementary material, Fig. S5), ANIDAGIGGQ and GIIQPQQPAQL). However, when the proteomics data were searched against the GluPro database the three diagnostic peptides were identified but were found to be present in numerous other γ-gliadin protein accessions (Fig. 4), and therefore were not classified as unique peptides to protein #P21292. The same phenomenon was observed for the other three gluten proteins (#P08489, P08488 and #P10386) identified in the Viridiplantae searched mass spectrometry data. All protein accession identified when proteomics data was searched using the Viridiplantae database and GluPro database is shown in Table S5, Table S6 respectively.

**Fig. 5:**
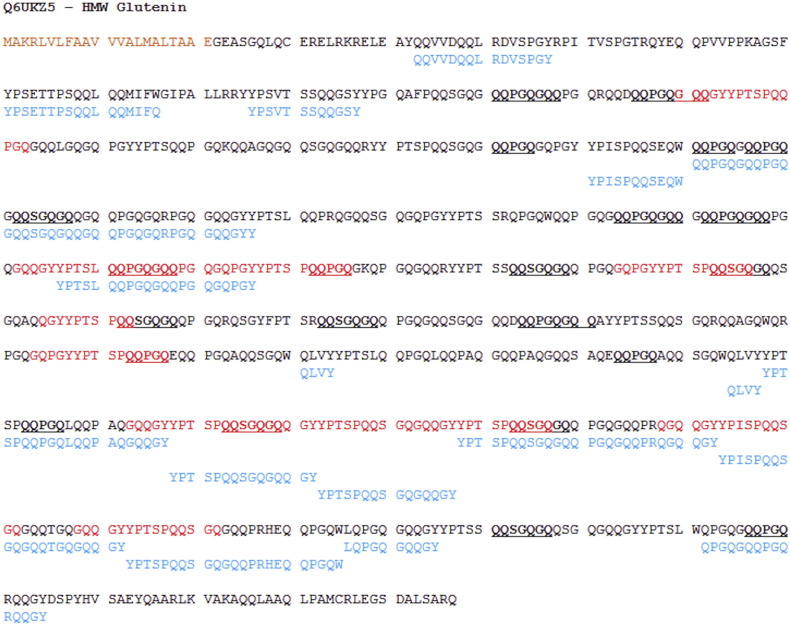
Protein sequence for HMW glutenin subunit Q6UKZ5: with mapped coeliac toxic regions from the Allergen Online database shown in the sequence as red, IgE epitopes shown by underlined text, and peptides identified in the experimental MS data shown below the protein sequence in blue. The signal peptide of the protein is shown in orange.

Mapping the coeliac toxic motifs onto proteins identified in the GluPro MS data, showed that the peptides identified often cover the coeliac toxic motifs. An example of this is shown using HMW glutenin subunit protein accession #Q6UKZ5 (Fig. 5) which contains 16 unique (i.e. different) coeliac toxic motifs which cover 20.75% of the 795 amino acids in the protein sequence. This shows that 165 residues constitute “coeliac toxic regions” (Fig. 5). Approximately 45% of these coeliac toxic regions are covered by peptides observed in the MS data shown in blue on Fig. 5. In addition, IgE epitopes [37], [39], [41] were also mapped to the protein sequence to show identify peptides relevant to IgE-mediated allergy to wheat.

## 4 Discussion

The significant reduction in the number of sequences remaining in the curated sequence database (2.6%) compared to the total number of sequences downloaded highlights the excessive redundancy and mis-annotations present in the un-reviewed UniProt database. There are also shortcomings in the reviewed UniProt database since the number of gluten protein accessions from bread wheat (*T. aestivum*) is small and not representative of the diversity of gluten protein sequences. This means care must be taken when reviewing proteomic MS data mined using such databases since there is a high probability of proteins being identified from plant species, such as grasses, which are closely related to bread wheat. This concern is supported by a comparison between the complete reviewed Viridiplantae database (37,577 sequences 14.05.2015) and the GluPro database, which identified few gluten protein species using the Viridiplantae database, the majority of which were fragments or derived from other species such as rye (*Secale cereale*) and the diploid wild grass *Aegilops tauschii* which is the donor of the D genome of bread wheat. Furthermore, with regard to mining the reviewed Viridiplantae database, the small number of gluten protein sequences present in such a large database meant that peptides identified as unique to a particular protein species, were in fact present in entire subgroups of gluten proteins and present in several related protein species. Such shortcomings emphasise the need for a curated gluten protein sequence database to support proteomics data analysis, as has been identified by others [50], [51].

Determining the number and distribution of coeliac toxic sequences across the GluPro database is relevant to its application in identifying relevant peptide targets for subsequent development of targeted multiple reaction monitoring experiments to quantify gluten proteins in food. It was observed that 98.6% of the coeliac toxic motifs had a minimum sequence length of nine amino acids, which is likely required to elicit a toxic response through binding to the MHC class 2 receptors HLA-DQ2 and HLA-DQ8 associated with coeliac disease [52]. Twenty six DQ2 and DQ8 restricted T-cell epitopes recognised by CD4 + T cells have been identified as being relevant for triggering coeliac disease [52]. Currently there are 31 peptide sequences that are nine amino acids long which are defined as coeliac epitopes; however the number of coeliac toxic motifs containing these epitopes is an ongoing discussion [4].

The α-gliadins were shown to have consistently high contents of coeliac toxic motifs, irrespective of the approach taken. This observation is consistent with the widely held view that α-gliadins are the main proteins responsible for eliciting the non-IgE mediated reaction in coeliac disease [53], due to their high reactivity to anti-gliadin antibodies developed in coeliac disease [54], [55] and the characterisation of Fraser Fraction III which showed that it contained the α-gliadin toxic 33-mer [56].

However, analysis of the database also showed that all types of prolamins contain motifs and are therefore able to elicit a coeliac toxic response. For example, sequence coverage by coeliac toxic motifs showed that all gliadins may be coeliac toxic, with ω- and γ-gliadins having higher contents of coeliac-toxic motifs per sequence than α-gliadins. The LMW glutenin subunits are particularly interesting as one group contains no matched peptides to the 1016 coeliac toxic Allergen Online database. Mapping of measurement one for coeliac toxicity onto the LMW subunit phylogenetic tree showed that these sequences formed a distinct cluster. By contrast, whilst most HMW subunits contain numerous coeliac toxic motifs, they have a low density of motifs because of their length. The small proportion of HMW glutenins that have the highest number of coeliac toxic motifs (five) all originate from 1Dy subunits, showing the relative coeliac toxicity of the sequences differs between genomes. This identification of sequences which contain few or no coeliac toxic motifs could be exploited in breeding to develop types of wheat which are less coeliac toxic than current commercial cultivars [57]. The ability to combine experimental proteomics data with database analysis and knowledge of coeliac toxic motifs may allow peptides from coeliac toxic regions to be targeted for MS analysis as they are currently targeted with antibodies. Furthermore, the GluPro database has over 1000 fold more gluten proteins than present in the Viridiplantae database, resulting in more accurate and reliable annotations.

Unlike other databases, GluPro is specifically designed to be employed within proteomics data analysis and overcomes the issues of redundancy and mis-annotation for wheat gluten proteins. Future work will focus on expanding the database to include other wheat species defined as containing gluten (such as *Triticum spelta* and *Triticum durum*), and to incorporate data from currently available partial genome sequences (notably from the standard genotype Chinese Spring). Expanding the GluPro database to include other wheat species and other gluten containing cereals consumed in food (such as barley and rye) is also crucial to support the development of a legally recognised analytical detection method using protein mass spectrometry. The observations from the study are also relevant to the wider arena of plant proteomics, and highlight the need for curated protein sequence databases any plant species of interest for mass spectrometry identification and quantification.

## 5 Conclusion

One of the main purposes of many proteomics analyses is to identify as many proteins as possible, whilst ensuring the identifications are of high quality. However, in wheat this is limited by the lack of a full genome sequence in a form suitable for proteomics analysis. We have therefore provide a curated database of wheat gluten protein sequences which can be used to identify the presence of sequences related to coeliac toxicity and IgE-mediated allergy in food products. The database is provided in FASTA format (10.15127/1.308603) and is tailored to mining proteomics data.

The following are the supplementary data related to this article.

**Fig. S2:**
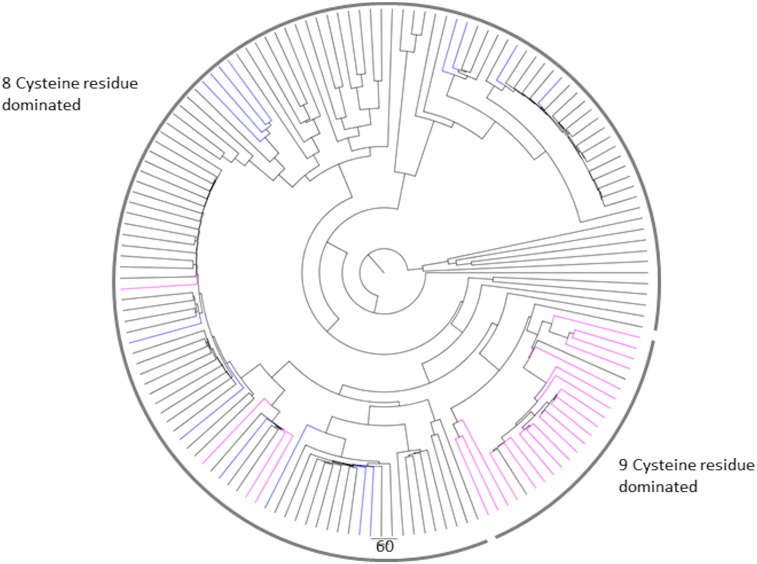
Phylogenetic tree of the 154 gamma gliadins, coloured by the number of cysteine residues in the sequence, with 7, 8 and 9 Cysteine residues represented in blue, black and pink respectively.

**Fig. S3:**
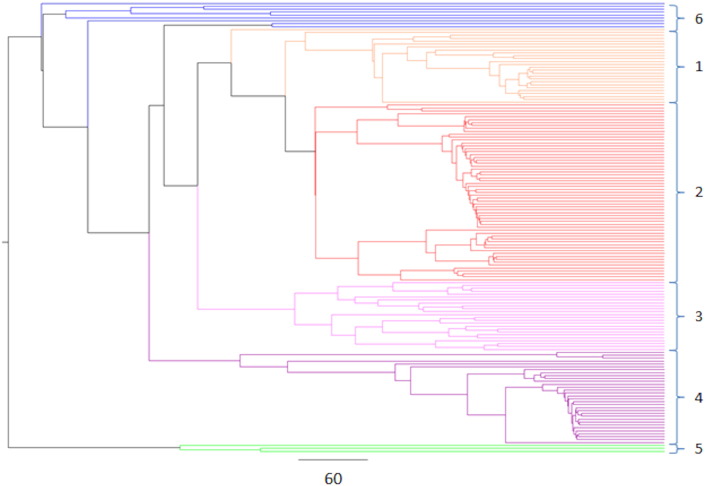
Phylogenetic tree of the 154 gamma gliadins, coloured by the clustering groups observed, which is expected to be due to the distinctive peptides observed in the repetitive domain. Group 1 – nonapeptide sequence PQQPFQLQPQ at residue 90. Group 2 – QPQQQFIQPQQ sequence at residue 90. Group 3 – QQPQQQFPQQPQQPFP sequence. Group 4 – QQTYPHQPQQQFPQTQQPQQPFPQP sequence.

**Fig. S4:**
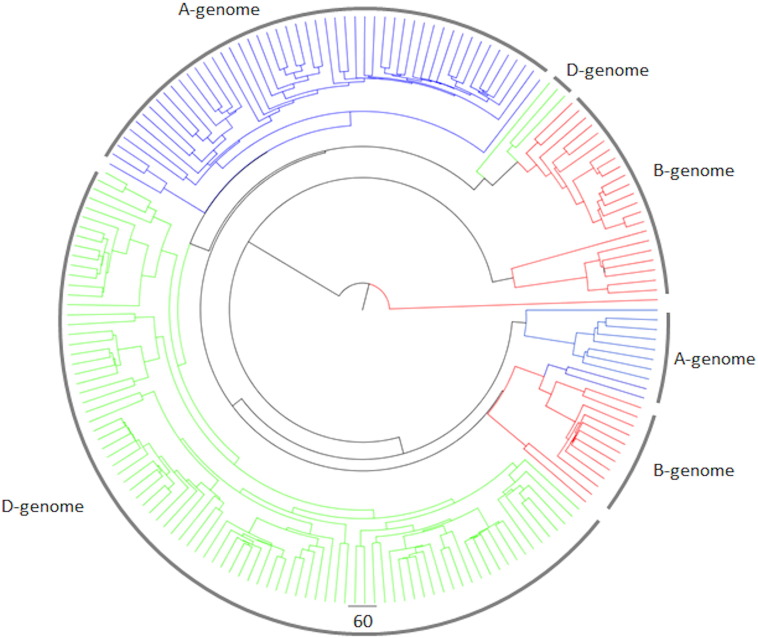
Phylogenetic tree of the 185 alpha gliadin, coloured by the genome origin of the sequence A, B and D coloured blue, red and green respectively.

**Fig. S5:**
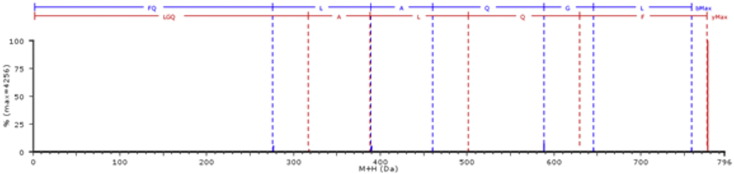
MS/MS fragmented spectra of the peptide FQLAQGL of the gamma gliadin protein P21292 identified when the processed proteomics data was searched against the complete reviewed viridiplantae database. The parts marked in blue and red represent the b and y ions respectively. The percentage identity is shown on the y-axis, with the mass of the protonated ion on the x-axis. The peptide has a peptide score of 6.2407.

Table S1 Table to show the NCBI accessions taken of the reviewed Wheat Gluten sequences taken from Wheat: Chemistry and Technology, and their corresponding UniProt Accessions. The resulting numbers of sequences from each BLAST search are shown in column D. Table S1

Table S2 Table to show the gluten protein group and the corresponding further classification and the number of accessions attributed to each classification. The UniProt accessions and the corresponding three measurements for coeliac toxicity are listed. Table S2

Table S3 In-house compiled list of gluten IgE epitopes divided into the different gliadin and glutenin protein groups. Table S3

Table S4 Summary of the N-terminal sequence for each of the distinct LMW classifications and the number of sequences observed in each classification. Table S4

Table S5 Table of protein accessions and protein scores with corresponding unique peptides sequences and *m*/*z* identified for LC-MS data searched against the complete reviewed Viridiplantae database. Table S5

Table S6 Table of protein accessions and protein scores with corresponding unique peptides sequences and *m*/*z* identified for LC-MS data searched against the GluPro database. Table S6

## Conflicts of interest

Lee Gethings and James Langridge declare a financial relationship with Waters Corporation that could be perceived to influence or give the appearance of potentially influencing the work submitted as both as employed by the mass spectrometry vendor used in this paper. Michael Bromley works within a consulting capacity for Synergy Health PLC providing analytical services, including allergen analysis, to the food industry. The other authors declare that the research was conducted in the absence of any commercial or financial relationship that could be construes as a potential conflict of interest.

## Transparency document

Transparency document Image 1
