# Supplementary material for: A curated gluten protein sequence database to support development of proteomics methods for determination of gluten in gluten-free foods
Source: J Proteomics. 2017 Jun 23;163:67–75. doi: 10.1016/j.jprot.2017.03.026 (PMC5479479; doi:10.1016/j.jprot.2017.03.026)
Supplement: Table S4 — Summary of the N-terminal sequence for each of the distinct LMW classifications and the number of sequences observed in each classification. [file mmc4.docx]

**Table S4: Summary of the N-terminal sequence for each of the distinct LMW classifications and the number of sequences observed in each classification**

| Nomenclature | N-Terminal Sequence | Number of Sequences |
| --- | --- | --- |
| LMW-s | M E N S H I P G L | 31 |
| LMW-i | I S Q Q Q | 30 |
| LMW-m_1_ | M D T S C I P | 13 |
| LMW-m_2_ | M E T S H I P | 36 |
| LMW-m_3_ | M E T S R V | 42 |
| LMW-m_4_ | M E T R C I P | 37 |
| LMW-m_5_ | M E T S C I | 35 |
